# Reassessing fluid creep: early targets for intervention in the PICU

**DOI:** 10.1038/s41390-025-04531-x

**Published:** 2025-10-24

**Authors:** Kelli M. Paice, Jennifer Kaplan

**Affiliations:** 1 Division of Critical Care Medicine, Department of Pediatrics, Penn State Health Children’s Hospital, Penn State College of Medicine, Hershey, PA USA; 2 Division of Critical Care Medicine, Department of Pediatrics, Cincinnati Children’s Hospital Medical Center, University of Cincinnati College of Medicine, Cincinnati, OH USA

We see it happen all the time to our patients – while our attention as intensivists is appropriately triaged to problems like impaired perfusion in the early resuscitative phase of pediatric critical illness, we often find ourselves 1–2 days later examining an edematous child and weighing (both figuratively and sometimes literally) if they are ready to begin their nearly inevitable course of diuretics. The development of post-resuscitation fluid overload (FO) has become so common that a term has been coined for it. The term “fluid creep” which was initially so named in the trauma and burn literature, has been echoed across other areas of critical care medicine.^1^ “Fluid creep” is almost invariably followed by an attempt to “pay the water bill” and achieve return to euvolemia with use of diuretics or kidney replacement therapies. But is this sequence truly inevitable, and should FO be tolerated?

Historically FO has been viewed as both a result of critical illness pathophysiology and also as a necessary side effect of critical resuscitative and interventional measures.^2^ However there has been growing evidence to suggest that increasing levels of FO are associated with negative clinical outcomes– including increased duration of mechanical ventilation, acute kidney injury, and mortality.^3–5^ The most recent pediatric Surviving Sepsis Guidelines even advise caution in using more than 40-60 ml/kg in fluid boluses during initial resuscitation, in part due to risk of development of FO.^6^ Further, there is growing interest in critical illness physiology, fluid status and associated volume of distribution change which may affect the pharmacokinetic of medications,^7^ and even the accuracy of laboratory measurements like serum creatinine.^8^

There are numerous factors that make children especially at risk for FO. Young children often have limited glycogen stores and thus require a steady glucose infusion rate, necessitating dextrose fluid administration while NPO. They have presumed higher insensible fluid losses, placing providers on guard for avoiding inadequate maintenance hydration. Fluids for line patency and default concentrations of continuous infusions are proportionally larger volume burdens for smaller patients, especially when degree of illness necessitates polypharmacy and multiple lumens of vascular access.

However, if it is well demonstrated that FO, especially at the extremes, is associated with negative outcomes, how then in the complex and dynamic environment of care for the critically ill child, are providers to focus their attention with avoidance and intervention in mind? This is one of the key questions addressed by Rao and colleagues in their study “Association between early fluid overload and clinical outcomes in a pediatric ICU,”^9^ which seeks to not only provide further evidence as to association between the FO phenomenon and negative PICU outcomes, but moves a step further to investigate the root causes of FO in order to propose targets for possible intervention.

In this large and well-designed retrospective observational cohort study of pediatric medical and surgical ICU patients with length of stay of least 48 hours, over 3000 patients were stratified at 48 hours by FO status into groups of “mild” ( <5%), “moderate” (5-10%), and “severe” (10%) FO. The severe FO group made up 19% of the overall cohort. Unsurprisingly, the authors found that the most fluid overloaded group had higher pSOFA scores, younger age, smaller body weight, and higher relative requirement for resuscitative fluids than the other two groups. Both moderate and severe FO was associated with increased ICU LOS, and severe FO was associated with increased rates of AKI and fewer ventilator-free days at 28-days, though it is unclear how well the study was powered to detect these or other outcome differences.

While early vasopressors use has been proposed and explored as a method to limit fluid exposure, in this study though severe FO group had the highest rate of vasopressor use (28%), they simultaneously still demonstrated the highest proportion of resuscitative fluid-intake (75%). These findings suggest that perhaps vasopressor use is not functionally as fluid sparing as we would like to believe, especially in a group with higher multiorgan dysfunction. Similarly, Rao et al. found that the severe FO group had the lowest urine output, even though all 3 groups were prescribed similar diuretic exposure. So if increased vasopressor use does not correlate with less need for resuscitative fluid, and diuretics prove relatively less efficacious for the most severely fluid overloaded patients, how then do we *prevent* the fluid overload in the first place?

To address this question, the authors reviewed the fluid exposures of patients in each of the FO groups and pragmatically divided fluid intake types into 3 categories: “non-modifiable” (resuscitative fluid, blood product and enteral nutrition), “modifiable” (maintenance fluids, carriers and flushes), and “potentially modifiable fluids” (parenteral nutrition, continuous and intermittent medications). They found that patients with severe FO had dramatically higher median maintenance IVF intake over 48 hours than the other two FO groups, and that mIVF were by far the largest volume of fluid administered of any subtype. In comparison, median resuscitation fluid input for the severe FO group was only 20 ml/kg, while the medians for the mild and moderate FO group were both 0 ml/kg over this time period. Continuous and intermittent medication fluid intake was similarly highest in the severe FO group, but was not as dramatically disparate from the mild and moderate FO groups.

Finally, the authors examined the dynamic nature of fluid balance in the ICU by tracking FO over the first 7 days of ICU stay, and comparing types of fluid intake predominating at each phase, as shown in Figure 2. While non-modifiable resuscitation peaked on day 1 and declined, and non-modifiable enteral nutrition increased in relative contribution to fluid intake around day 2, modifiable maintenance IVF showed ongoing prominence across all 7 study days.

One major limitation of this study, as acknowledged by the authors, is the use of the Holliday Segar formula to calculate assumed maintenance fluid needs in their analysis^10^ - however this method is also commonly used for general clinical decision making around maintenance fluid rates in critically ill children. These formulas are simply not designed for critically ill patients who may need far more or far less fluid, depending on their type and stage of illness. Another notable choice by the authors was to include patients that had negative fluid balance at 48 hour in the <5% “mild” fluid overload group, which Figure 1 suggests may have been nearly 25% of this cohort. Isolation and evaluation of this group may have provided unique insights into their outcome markers and the volumes/types of fluids that yielded this negative balance, or may have made the differences between the mild FO group and the moderate and severe FO groups less significant.

Despite these limitations, this study highlights both fluid types to target, and time-windows of opportunity to potentially prevent the development of FO in critically ill children. While it could be argued that some of the fluid types they list as “potentially modifiable” may be better described as “partially modifiable” – i.e. it may be difficult to limit necessary medications or inadvisable to delay parenteral nutrition in certain patients at different time-point in their illness, we may be able to adjust volume or concentrations of these types of fluid to limit overall exposure. However what is clear is that maintenance IV fluids, especially by day 2 of illness and beyond, represent a large burden of fluid intake for our patients, and thus represent the lowest hanging fruit to reduce overall fluid exposures. Methods to control fluid balance in this regard might include use of a total fluid goal or total hourly fluid rate orders, or more intentional maintenance fluid rate reduction and real-time fluid status awareness.
